# Construction Notes

**Published:** 1893-07-15

**Authors:** 


### CONSTRUCTION NOTES.

Addition to Cookridge Hospital.—It is proposed to erect a new wing to Cookridge Convalescent Hospital, to be called "The Canon Jackson Wing," in memory of the late Canon Jackson. The proposed addition will provide fifty more beds to those already existing. The preparation of the plans has been entrusted to Mr. Walter A. Hobson, of Leeds. The whole of the new wing is practically devoted to the provision of additional sleeping accommodation, with the exception of a portion which has been utilised for enlarging the existing dining hall, and providing a men's new day-room. Externally, this extension will be carried out in harmony with the present building, and will extend the present front 60 feet to the east, and will run back to a depth of 72 feet. On the ground floor will be located the men's day-room, which will be 34½ by 23 feet. This floor will also contain a staircase hall, 12 feet in width and 22 in length, giving access on either side to dormitories for eight or 12 beds respectively. On the first floor a men's dormis tory, 34 by 24 feet, and with accommodation for 12 beds, i- placed over the men's day-room, and at right angles to it there are other two dormitories exactly similar to these on the floor below. The second floor is practically a repetiton of the first, a staircase running from the ground floor to the top storey gives access to the dormitories which are placed on either side of it, and which can be ventilated into it. The new wing is L-shaped, and the lavatories, which are provided for every floor, are placed in the angle of the building. The existing men's day-room will be enlarged into a dining hall 57 by 23 feet in size. The open path at the rear of the building will be roofed with glass, and will be used as a winter garden, besides which it will serve to connect the men's smoking-room with the new wing and the other parts of the building. The dormitories, day-room, and entrance hall will be warmed in winter with hot water from the already existing boiler. The contractors for all trades are Messrs. Cross and Carter, of Leeds, Messrs. Thomas Green and Sons having been entrusted with the carrying out of the heating apparatus. The cost for the entire extension, when completed, will be about £4,000.

